# Temporal genetic changes in *Plasmodium vivax* apical membrane antigen 1 over 19 years of transmission in southern Mexico

**DOI:** 10.1186/s13071-017-2156-y

**Published:** 2017-05-02

**Authors:** Alejandro Flores-Alanis, Lilia González-Cerón, Frida Santillán, Cecilia Ximenez, Marco A. Sandoval, René Cerritos

**Affiliations:** 1 División de Investigación, Facultad de Medicina, Universidad Nacional Autónoma de México, Ciudad de México, 04510 Mexico; 2 Centro Regional de Investigación en Salud Pública, Instituto Nacional de Salud Pública, Tapachula, Chiapas 30700 Mexico; 3 Departamento de Medicina Experimental, Facultad de Medicina, Universidad Nacional Autónoma de México, Ciudad de México, 06729 Mexico

**Keywords:** *Plasmodium vivax*, Apical membrane antigen 1, Southern Mexico, *pvama1*_*I-II*_, Genetic structure, Evolution, Allelic frequency, Temporal haplotype network

## Abstract

**Background:**

Mexico advanced to the pre-elimination phase in 2009 due to a significant reduction in malaria cases, and since 2000, *Plasmodium vivax* is the only species transmitted. During the last two decades, malaria transmission has been mostly local and isolated to a few regions. It is important to gain further insights into the impact of control measures on the parasite population structure. Hence, the aim of the current study was to determine detailed changes in *P. vivax* genetic diversity and population structure based on analysing the gene that encodes the apical membrane antigen 1 (*pvama1*). This analysis covered from control to pre-elimination (1993–2011) in a hypo-endemic region in southern Mexico.

**Results:**

The 213 *pvama1*_*I-II*_ sequences presently analysed were grouped into six periods of three years each. They showed low genetic diversity, with 15 haplotypes resolved. Among the DNA sequences, there was a gradual decrease in genetic diversity, the number of mixed genotype infections and the intensity of positive selection, in agreement with the parallel decline in malaria cases. At the same time, linkage disequilibrium (R^2^) increased. The three-dimensional haplotype network revealed that *pvama1*_*I-II*_ haplotypes were separated by 1–11 mutational steps, and between one another by 0–3 unsampled haplotypes. In the temporal network, seven haplotypes were detected in at least two of the six-time layers, and only four distinct haplotypes were evidenced in the pre-elimination phase. Structure analysis indicated that three subpopulations fluctuated over time. Only 8.5% of the samples had mixed ancestry. In the pre-elimination phase, subpopulation P1 was drastically reduced, and the admixture was absent.

**Conclusions:**

The results suggest that *P. vivax* in southern Mexico evolved based on local adaptation into three “pseudoclonal” subpopulations that diversified at the regional level and persisted over time, although with varying frequency. Control measures and climate events influenced the number of malaria cases and the genetic structure. The sharp decrease in parasite diversity and other related genetic parameters during the pre-elimination phase suggests that malaria elimination is possible in the near future. These results are useful for epidemiological surveillance.

**Electronic supplementary material:**

The online version of this article (doi:10.1186/s13071-017-2156-y) contains supplementary material, which is available to authorized users.

## Background

*Plasmodium vivax* is the most prevalent malaria species in Latin America, the Middle East, South and Southeast Asia, Oceania and The Horn of Africa [1]. In these regions, more than 2.5 billion people are at risk [1], and approximately 13.8 million cases were reported in 2014 [2]. During the 2000–2014 period in Latin America, malaria cases gradually diminished from 1.2 million to 390,000 cases, representing a 74% decline. Argentina, Paraguay and Costa Rica are in the elimination phase, while Belize, Dominican Republic, Ecuador, El Salvador and Mexico are in the pre-elimination phase [2].

In Mexico, the annual number of malaria cases fluctuated between 20,000 and 130,000 in the 1980s. Since the late 90’s, the improvement of strategies and efforts by the malaria national control program has driven a continuous decrease in malaria incidence, with only *P. vivax* causing cases of autochthonous transmission. The Pacific coast is the location of almost all transmission, mainly near the boundaries between Mexico and Guatemala as well as between the states of Chihuahua, Sinaloa and Sonora, as well as Durango and Nayarit. In the former case, along the Mexican side of the border is the State of Chiapas, and transmission occurs at the pacific side and in the Lacandon rainforest [3, 4].

*Plasmodium vivax* is haploid in the human host [5], which facilitates the haplotype assembly when analysing human blood stages. It has been found that this species is indeed genetically diverse worldwide [6, 7] and that the haplotype frequency can be modified by evolutionary forces such as mutation, natural selection, migration and genetic drift [8–10]. The understanding of how these forces induce *P. vivax* populations to differentiate and expand in time and space [11] are relevant for epidemiological surveillance and essential for the design and evaluation of control and elimination strategies. To study the genetic diversity of *P. vivax*, different molecular markers have been used, including microsatellites [7, 12–15], mitochondrial genes [16–18] and genes encoding antigenic molecules [19–22]. Many blood stage antigens especially those involved in immune evasion are highly polymorphic; they evolve more rapidly than neutral markers and allow high resolution of parasite haplotypes [23].

The apical membrane antigen 1 (*ama1*) gene is located on chromosome 9 and encodes an integral membrane protein type 1, considered as a strong vaccine candidate (AMA1). This antigen has an ectodomain that plays a key role in the reorientation of the merozoite during the invasion of red blood cells [24, 25]. The ectodomain comprises three domains: domain I, domain II and domain III [26, 27]. In domain DI of *P. vivax*, most studied, higher nonsynonymous than synonymous nucleotide changes have been detected in isolates from distinct regions such as Asia [28–31], Oceania [22], the Middle East [32] and Latin America [33].

The *P. vivax* population is genetically unique in southern Mexico. By using microsatellite markers, three subpopulations related to mosquito specificity and geographical distribution were identified [12]. Accordingly, the ookinete Pvs25-28 polymorphism was associated with vector compatibility [34]. The icb5-6 blocks of *pvmsp1* revealed new hybrid lineages that diversified, possibly because of recombination between divergent haplotypes from South America and Asia [35]. In additional, the analysis of *Pvama1*_*I-II*_ in a limited number of samples from 2006–2007, exposed that this gene fragment had moderate genetic diversity and was found under balancing selection [36].

Population and evolutionary genetics provide knowledge about how selective forces are networking in any species. This knowledge is useful for evaluating the impact of control and elimination measures on the population structure of vector parasites. Hence, the aim of the current longitudinal study was to investigate changes in *P. vivax* diversity and population structure over 19 years, based on analysis of *pvama1*_*I-II*_ *,* in samples taken in southern Mexico. The study was from 1993 to 2011, a period comprising control and pre-elimination phases.

## Methods

### *Plasmodium vivax* samples

A total of 288 samples (105 of whole blood and 183 of blood smeared on filter paper) had been previously obtained from symptomatic patients, for about two-decade period since 1993 [37–39]. The *P. vivax* diagnosis was carried out at the laboratory facility of the Regional Center for Research in Public Health (CRISP-INSP). Patients lived in Jurisdiction VII of the State of Chiapas in Mexico, which comprises an area of 4644.07 km^2^ of tropical and template regions, with the altitude ranging from sea level to foothills up to 4,000 m above sea level. The number of *P. vivax* samples analyzed per year were as follows: 1993, *n* = 12; 1994, *n* = 25; 1995, *n* = 8; 1996, *n* = 4; 1997, *n* = 18; 1998, *n* = 18; 1999, *n* = 21; 2000, *n* = 17; 2001, *n* = 11; 2002, *n* = 19; 2003, *n* = 22; 2004, *n* = 20; 2005, *n* = 21; 2008, *n* = 22; 2009, *n* = 20; 2010, *n* = 17; and 2011, *n* = 13. DNA extraction was carried out with the QIAamp ® DNA Blood Mini Kit (Qiagen, Redwood City, CA, USA), following the manufacturer’s instructions.

### *Pvama1*_*I*-*II*_ amplification and sequencing

A DNA fragment comprising domains DI and DII (*pvama1*_*I*-*II*_) was amplified. The reaction mixture consisted of 5 μl of 10× reaction buffer (600 mM Tris-SO4 at pH 8.9 and 180 mM (NH_4_)_2_SO_4_, 2 μl of MgSO_4_ (2 mM), 1 μl of dNTPs (0.2 mM), 1.8 μl of each primer at 36 pM (PvPvama1F 5′-TCC AGC TGG AAG ATG TCC TG-3 ′ and Pvama1R 5′-CCG CCC TTT TCT CTA CAC AG-3′), 0.2 μl enzyme Platinum® *Taq*DNA Polymerase High Fidelity Invitrogen™ (1 U per reaction), and 2–4 μl of DNA. The final volume was adjusted to 50 μl. The PCR conditions were as follows: the first cycle at 95 °C for 5 min, followed by 35 cycles at 95 °C for 60 s, one cycle at 61 °C for 60 s, another at 72 °C for 75 s, and a final cycle at 72 °C for 10 min. The reactions were performed in a Thermal Cycler 2720 thermocycler (Applied Biosystems®, CA, USA). The samples that produced little or no visible PCR product were re-amplified with a nested PCR by using primers Pvama1F and Pvama1R2 5′-CGC AGG GAC ATT TGA TAC TCT CC-3′ and 1–2 μl of PCR product from the first PCR reaction.

The PCR-amplified products were separated by electrophoresis with agarose gels at 1%. For visualisation, 0.2 μg/ml ethidium bromide was added, and they were observed in an ultraviolet light chamber. The PCR products of approximately 1,100 bp were purified using the MinElute® PCR Purification Kit (Qiagen, Redwood City, CA, USA), according to the manufacturer’s instructions. The purified DNA samples were sequenced on an ABI 3730xl DNA Analyzer (ThermoFisher, Waltham, MA, USA) in the High-Performance Center in Genomics (Seattle, WA, USA).

The electropherograms of 24 samples showed double peaks, suggesting mixed genotype infections. Therefore, DNA fragments obtained from the first PCR were cloned by using the TOPO TA Cloning Kit with pCR® 2.1-TOPO (Invitrogen, Carlsbad, CA, USA). For each isolate, three to five different clones were sequenced.

The nucleotide sequences were revised by using the BioEdit version 5.0.9 Sequencing Alignment Editor Copyright © program and aligned with Clustal W [40], utilising the sequence of strain Sal I as a reference. The consensus sequences were deposited in the GenBank database (Accession numbers: KY094724–KY094901).

### Genetic analyses

The population genetic analysis was performed for all samples (*n =* 213) and for subgroups (defined by time periods). DnaSP v5.1 was used to determine the number of polymorphisms (S), haplotypes (h), and synonymous (*dS*) and non-synonymous (*dN*) changes, as well as the minimum number of recombination events (Rm), the haplotype diversity (Hd), genetic diversity (indexes π and θ), and linkage disequilibrium (LD) given by index R^2^. Moreover, to ascertain whether the observed diversity departs from neutrality, the difference between the substitution rates of nonsynonymous (*dN*) and synonymous (*dS*) nucleotide changes (*dN - dS*) was evaluated within populations by using the MEGA v6.0 program [41]. This estimation was based on the maximum joint likelihood reconstruction of ancestral states under the models of Muse-Gaut [42] and substitution of codons [43]. In addition, data from the neutrality test McDonald-Kreitman was calculated [44] with DnaSP v5.1. As the outgroup, a *Plasmodium cynomolgi* sequence was used (GenBank X66099.1) [45].

*Plasmodium vivax* haplotype networks were constructed to investigate their genetic relationships, which change over time. First, all the sequences from the parasites in southern Mexico were included to prepare the “master” haplotype network with the TCS 2.21 program. This network was the template to prepare the layers of the temporal network, one per period. The networks were piled up to produce a three-dimensional effect, thus facilitating a comparison of the relationships of the haplotypes and their frequency. The relationship between haplotypes was resolved through mutational steps, which allowed for the assignment of existing haplotypes to a common ancestor of the population [46].

STRUCTURE (v2.3.4) analysis was implemented to explore how local haplotypes were grouped into subpopulations, and if possible to visualise changes in the frequency of the defined subpopulations over time. This technique employs a Bayesian approach to establish the probability of the number of populations in a sample of sequences. The analysis was carried out for 50,000 iterations, followed by 100,000 Markov Chain Monte Carlo (MCMC), and all runs were based on the model of admixture [47]. Twenty replicates run with a K-value that ranged from two to six predicted the optimum value of K, and the probability LNP [D / K] was calculated [48]. The fixation index (*F*_ST_) was determined between parasite subgroups of southern Mexico (defined by time periods) and for parasites from distinct geographic origins using the program DnaSP v5.1.

Homologous sequences of *P. vivax* from different geographic sites were found at NCBI, GenBank: Venezuela (VNZ), EU346015–EU346087 [33]; Iran (IR) JF682785–JF682790 (Unpublished), JX624732–JX624760 [32]; Sri Lanka (SLK), EF218679–EF218701 [28], India (IND) EU282774–EU282822 [29], EF025187–EF025197 [49]; Papua New Guinea (PNG), KC702458, KC702402–KC702503 [22]; Thailand (THL) FJ784891–FJ785121 [30] and South Korea (SK) KM230319–KM230384 [31].

## Results

### *Plasmodium vivax ama1*_*I-II*_ from southern Mexico, 1993–2011

#### Polymorphism and MGI

One hundred and seventy-eight *pvama1*_I-II_ sequences comprising a fragment of 702 bp were obtained from 176 parasite isolates; eighty-four percent (*n* = 89) of whole blood samples (period 2002–2011), and only 47.5% (*n* = 87) of blood impregnated onto filter paper. The *pvama1*_I-II_ gene fragment was obtained from all samples from 1995 (*n* = 8) but none of the samples from 1996 (*n* = 4). For other years the proportion of samples with a high-quality sequence varied between 17 and 58%. Failure to amplify parasite DNA or to obtain a good quality sequence could be due to low parasitemia, and the quality of DNA preserved onto filter paper.

The electropherograms suggested only one haplotype (assigned as a single genotype infection) for 158 isolates, while 18 samples (10.33%) had more than one peak in at least one nucleotide position (suggesting more than one genotype per sample). The samples were re-sequenced and electropherograms reviewed by three examiners. The cloning resolved only for two samples, two haplotypes per each sample.

Thirty-five previously reported sequences (KP759815–KP759849) from 2006 and 2007 [36] were included in the analysis, which made 213 sequences.

### Genetic diversity

The16 polymorphic sites were dimorphic. There were ten changes in the DI domain and six in the DII domain (Additional file 1: Table S1). The whole gene fragment (DI-DII) resolved fifteen haplotypes (H1-H15). Only one nucleotide change existed between H1 and H7, and one between H8 and H11. Haplotype H7 was also present in a single genotype infection. Seven polymorphisms were exclusive to southern Mexico, as previously described [36], and only two other nucleotide changes were detected in a single parasite from 2010 (T583A and A911G). Eight of 15 haplotypes were exclusive for southern Mexico (H1, H4, H7, H8, H9, H10, H14 and H15) (Additional file 2: Figure S1). At least one of the other haplotypes was previously reported in Venezuela [33], Iran [32], India [29, 49], Sri Lanka [28], Thailand [30] or South Korea [31] (see also Additional file 2: Figure S1).

For *pvama1*_*I-II*_ in southern Mexico, the overall nucleotide (π) and genetic diversity (θ) were low, being 0.0067 ± 0.0002 and 0.0038 ± 0.0009, respectively. These values are similar to those reported for a sample of 35 parasites from 2006–2007 [36], and above those calculated for parasites from Venezuela [33] or South Korea [31], both sites of low transmission [2]. Other geographic sites with high transmission had greater genetic diversity (0.0073–0.0101), including Iran [32], India [29, 49], Sri Lanka [28], PNG [22] and Thailand [30] (Additional file 3: Table S2). Parasites from southern Mexico had the lowest haplotype diversity (0.734), with their Rm value being similar to that found in parasites from Venezuela and South Korea (Additional file 3: Table S2).

### Natural selection

The *dN - dS* value was positive (1.527; *P* > 0.05) for southern Mexico, as it was at the global level (0.806), in neither case reaching significance. There were similar values for other geographic sites, except SLK (2.154, *P* <0.05). However, the McDonald-Kreitman test was positive and highly significant at the global and local level for all geographic sites herein analysed (Table 1).

**Table 1:** Natural selection tests on *p*v*ama1*_*I-II*_ among *P. vivax* populations of different geographic origins

Country	Polymorphic changes within *P. vivax* populations		*dN-dS* value^a^	*P*	Fixed changes between species^b^		McDonald-Kreitman (NI)	Fisher’s exact test (*P-v*alue)
	Synonymous	Nonsynonymous			Synonymous	Nonsynonymous		
SMX	1	15	1.510	0.13	53	25	31.800	< 0.000001
VNZ	2	9	0.821	0.41	54	28	8.679	0.00591
IR	4	24	0.844	0.40	51	24	12.750	< 0.000001
SLK	2	21	2.154	0.03	53	24	23.188	< 0.000001
IND	7	23	1.012	0.31	53	24	7.253	0.00002
PNG	4	15	1.725	0.09	51	24	12.750	0.00001
THL	7	19	-0.071	0.94	50	21	8.333	0.00002
SK	10	16	-0.425	0.67	51	25	3.264	0.01934
Total	20	44	0.836	0.40	45	20	4.950	0.00002

^a^ *dN-dS,* the difference between the rate of nonsynonymous and synonymous mutations

^b^ *Plasmodium cynomolgi* was used as the outgroup species (GenBank: X66099.1)

*Abbreviations*: *NI* Neutrality Index (significance at 95%), *SMX* southern Mexico, *VEN* Venezuela, *IR* Iran, *SLK* Sri Lanka, *IND* India, *PNG* Papua New Guinea, *THL* Thailand, *SK* South Korea

### Linkage disequilibrium

The LD coefficient R^2^ for southern Mexican parasites (0.5103) was moderate (Additional file 3: Table S2) compared to the elevated values at sites with the low transmission. Contrarily, R^2^ was low at sites showing high transmission.

### *F*_ST_*values*

*Plasmodium vivax* from distinct geographical origins had from moderate to high values. Southern Mexican parasites had *F*_ST_ values from 0.1267 to 0.4601 when compared to other regions. Between southern Mexico or Venezuela and Iran, moderate *F*_ST_ values of 0.1267 and 0.1605 were obtained. Between southern Mexico and Venezuela, the value was higher (*F*_ST_ = 0.2251, *P* < 0.001). The greatest *F*_ST_ values were between South Korea and other sites (0.3654 to 0.6054). Moreover, *F*_ST_ values between Iran, India and Sri Lanka were close to zero (Additional file 4: Table S3).

### Temporal genetic analysis of *P. vivax ama1*_*I-II*_

**Fig. 1:**
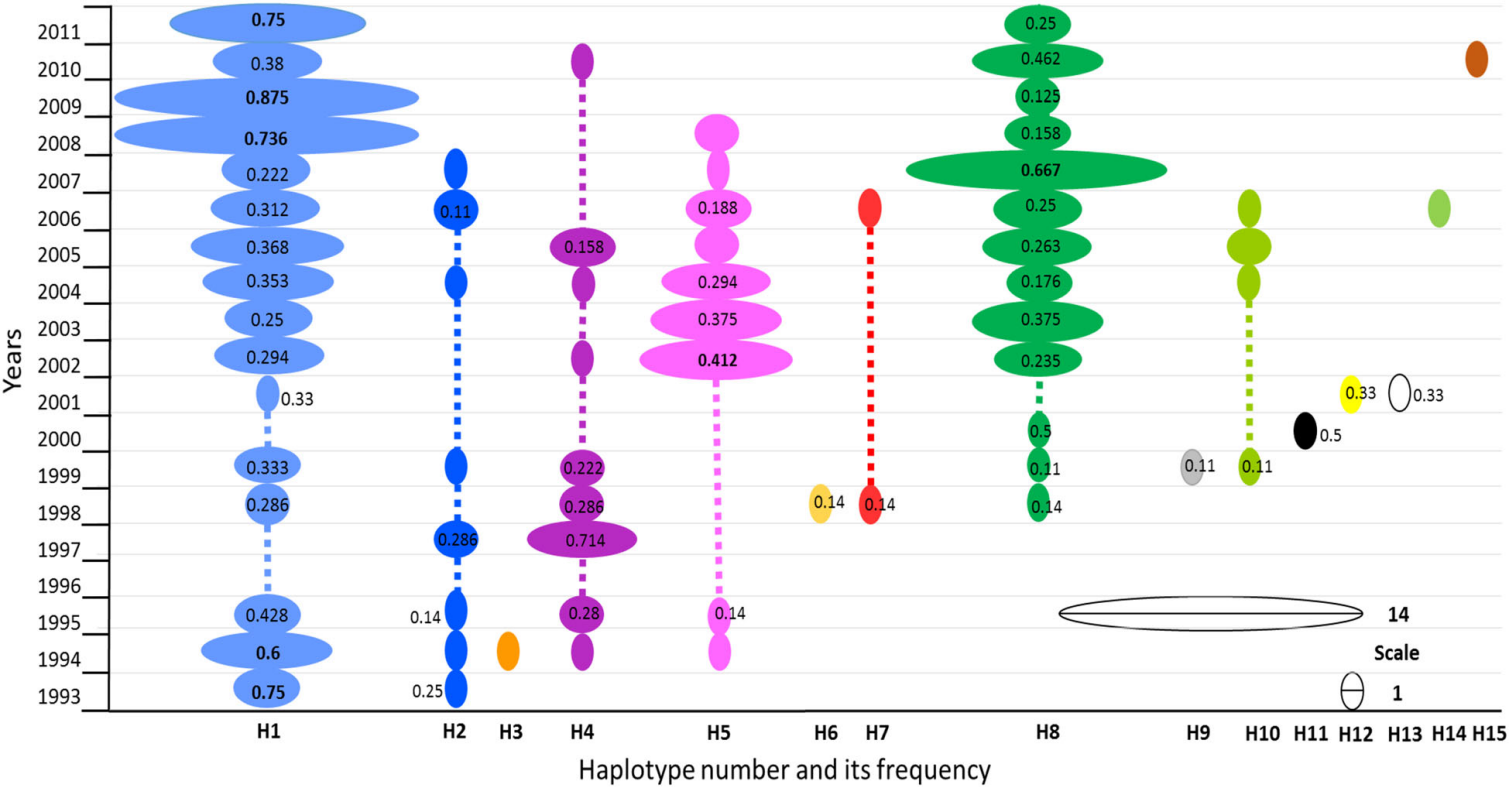
Yearly distribution of *p*v*ama1*_*I-II*_ haplotypes in southern Mexico during 19 years. Of 213 DNA sequences, 15 haplotypes were resolved (denominated H1-H15). The proportion of each haplotype in each year is represented by the size of the oval (only those representing ≥ 0.10 are indicated). Some haplotypes were highly frequent in a particular period and existed in samples collected from different years. There were variations in haplotype frequency. It is possible that certain haplotypes alternated with one another. For instance, H1 was present for almost the entire study, having an increased frequency during 2008–2009 and in 2011. This haplotype seemed to alternate with H8, which had a greater proportion in 2007 and in 2010. No samples from 1996 were tested

The number of sequences obtained per year varied from two to nineteen. Figure 1 shows the proportion of each haplotype found per year. There were important changes in haplotype frequency. For example, H8 was at a proportion of 0.667 in 2007, followed by its reduction to 0.158 in 2008. Contrarily, H1 increased to predominate in 2008 and 2009. In 2002, H5 was present at a higher frequency than H1 and H8. These sequences were grouped into six consecutive 3-year time periods, each including a minimum of 20 sequences: 1993–1995 (*n* = 21), 1997–1999 (*n* = 23), 2000–2002 (*n* = 22), 2003–2005 (*n* = 52), 2006–2008 (*n* = 54) and 2009–2011 (*n* = 41).

The highest proportion of samples presumably carrying mixed genotype infections (MGI) was determined for 1993–1995 and 1997–1999, resulting in a value of 23.1 and 47.8%, respectively. The percentage of mixed genotype infections dropped to 4.5 and 2.4% for 2000–2002 and 2009–2011, respectively. In only two cases, the cloning resolved two haplotypes per sample: sample number 8998 included haplotypes H1 and H7, and sample number 2500 had H8 and H11. For another 12 isolates, only one haplotype per sample was resolved, while four other isolates were not possible to clone.

**Fig. 2:**
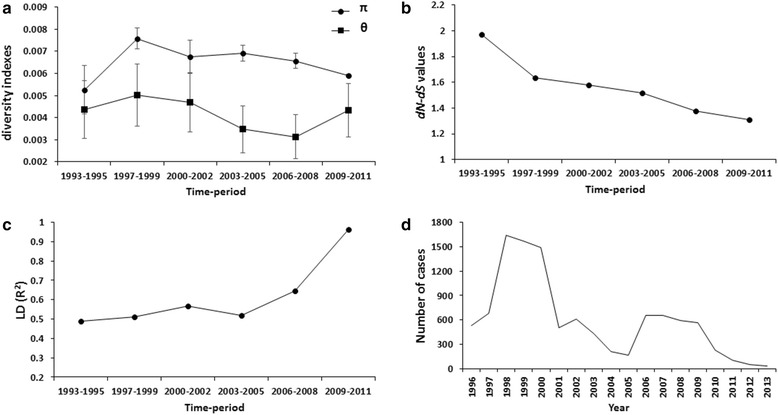
Temporal genetic changes for *P. vivax ama1*_*I-II*_ in southern Mexico from 1993 to 2011. **a** Nucleotide diversity (π) and genetic diversity (θ). **b** Linkage disequilibrium given by R^2^. **c** Natural selection by *dN-dS* values. **d** Annual incidence of malaria according to confirmed cases in the 1993–2011 period within Jurisdiction VII of Chiapas

The genetic parameters varied over time. In the samples from 1993–1995, the nucleotide diversity was the lowest (π = 0.00526). Then in the 1996–1999 period there was an increase in this π value (0.00758) (Fig. 2a), which coincided with the significant rise in malaria cases from 1998 to 2000 (Fig. 2d), likely as the result of the climactic event “El Niño”. Subsequently, a reduction in both malaria cases and genetic diversity took place during 2000–2002, followed by a greater decline in malaria cases (although genetic diversity is remaining the same) during 2003–2005. Despite the rise in malaria cases during 2006–2008, the nucleotide diversity (π) continued to diminish through the 2009–2011 period (0.00591 ± 0.00007). The theta value was consistently much lower than pi. In contrast, the LD index R^2^ underwent a gradual increase from 0.4886 to 0.9614 (Fig. 2b). The *dN - dS* value was positive and gradually decreased from 1.971 to 1.313, but was not significant (Fig. 2c). Figure 2d shows the number of confirmed malaria cases per year, compiled by the Sanitary Jurisdiction VII in the State of Chiapas, Mexico. The number of cases was high during 1998–2000, and then a sudden drop was registered. A second peak was observed in the 2006–2009 period, followed by a sustained decline after that. In this Jurisdiction, only 101 malaria cases were reported in 2011.

### Haplotype networks and temporal changes in pvama1_I-II_ allelic frequencies

**Fig. 3:**
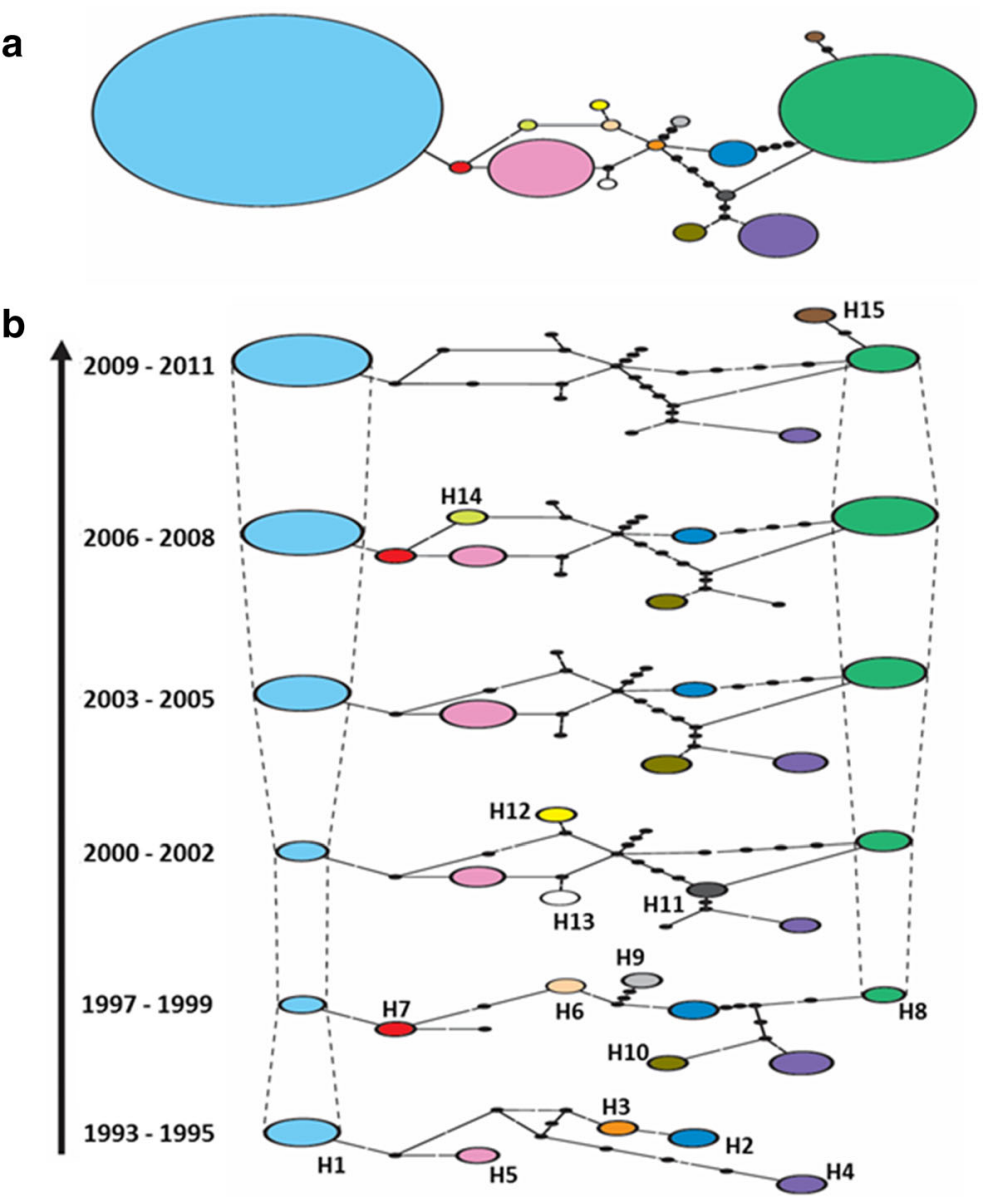
Temporal haplotype network of *P. vivax ama1*_*I-II*_ in southern Mexico. **a** The master network for the entire period (1993–2011). **b** Haplotype networks in a structure of six layers; the master network was used as a template and each layer represented a three-year period. Each color corresponds to a haplotype (H1-H15) and the size of the ovals is proportional to the number of isolates which share the same haplotype (except for H1 and H8; 0.5 magnification). Solid lines connect the haplotypes; black dots represent the number of mutational steps between haplotypes (including those sampled or extinct). Only the haplotypes detected in each time layer are shown in color

The master network showed that the 15 haplotypes from southern Mexico were separated by one to twelve mutational steps, and two sampled haplotypes by one to four mutational steps. The most frequent haplotypes, H1 and H8 at 42.9 and 24%, respectively, were highly divergent, being separated by at least nine mutational steps. Haplotype H1 was two mutational steps from H5 (13.6%) and two to eleven mutational steps from other low-frequency haplotypes (mostly detected in a single isolate). On the other hand, H8 was four mutational steps from H2 (4.2%) and H4 (8.5%), and two mutational steps from H15, which was found in a single isolate. The network also revealed two loops, suggesting recombination events between haplotypes H2, H3, H8 and H11, as well as between haplotypes H3, H5, H6, H7 and H14 (Fig. 3a).

Seven of 15 *pvama1*_*I-II*_ haplotypes (46.7%) were present in more than one period. Another eight haplotypes were unique, and six of these were observed from 1998 to 2001, the period with the maximum number of reported malaria cases (Fig. 3b). There were five divergent haplotypes (H2, H4, H5, H7 and H10) from the beginning to the end of the study. Though H8 was abundant from 2002 on, it was not encountered in the samples during 1993–1995. Haplotype H5, closely related to H1 and H7 and detected in the 1993–1995 period, was not found immediately after the climactic event “El Niño” (1997–2001) but was very frequently observed from 2002 to 2008. H2 and H4, which were separated from H8 by five mutational steps and from each other by nine mutational steps, had similar distribution overtime. Based on the haplotype network for global parasites, with 104 varieties, southern Mexico has three lineages that are closely related to haplotypes from Venezuela, India, Iran and South Korea (Additional file 2: Figure S1).

### Structure analysis

**Fig. 4:**
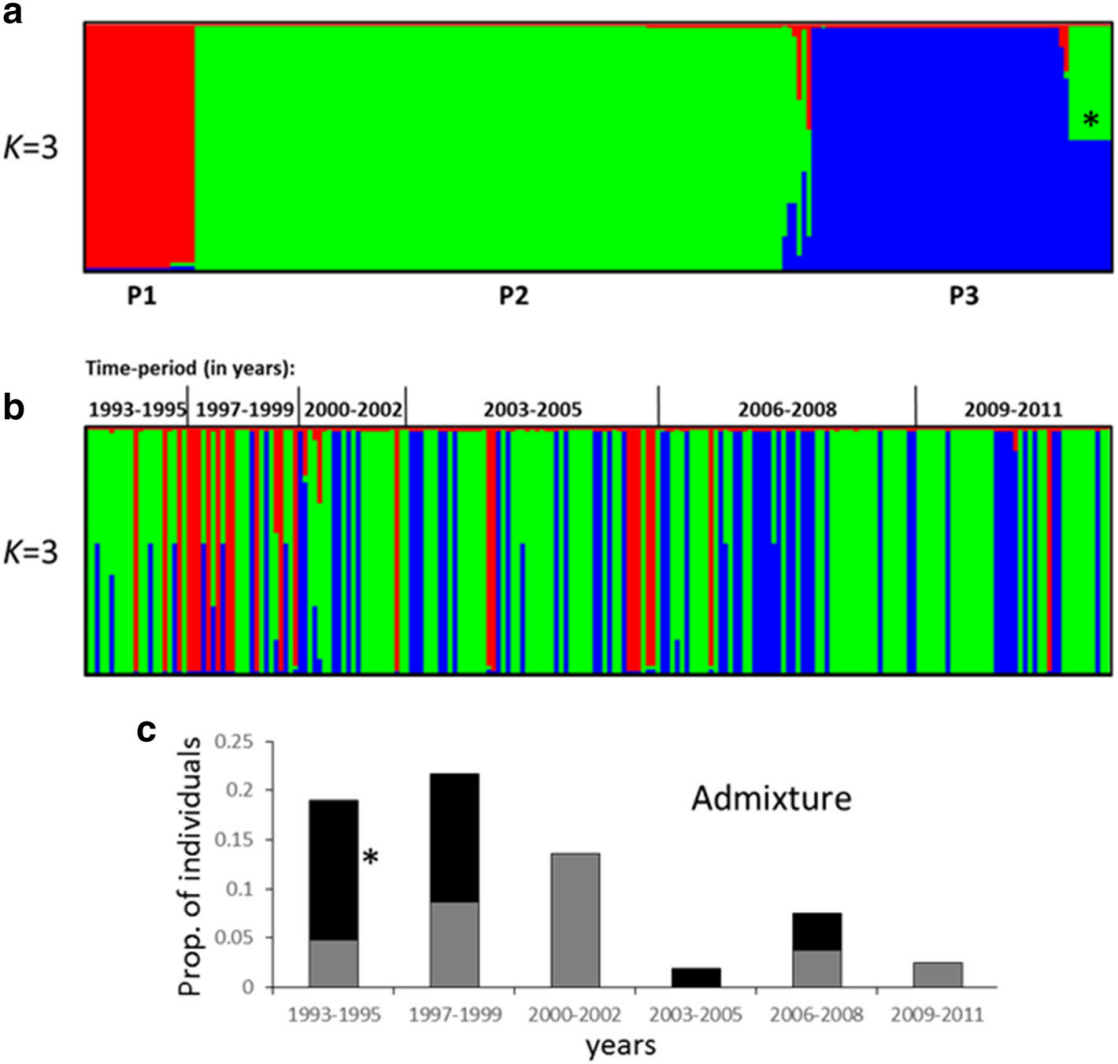
Population structure of *P. vivax ama1*_*I-II*_ in southern Mexico. The subpopulations are represented by different colors (K = 3): *red* (P1), *green* (P2) and *blue* (P3). A vertical bar represents each subject. **a** Resolution of the three subpopulations resulting from Bayesian cluster analysis. **b** Individuals are chronologically ordered, and periods are indicated. **c** The frequency of individuals with mixed ancestry is represented; one frequent type of admixed genotype (P2/P3*) is shown in *black* and other admixed types in *grey*

The 15 haplotypes from southern Mexico were grouped into three subpopulations (P1, P2 and P3) (Fig. 4a). P2 comprised ~ 57%, followed by P3 at ~ 24% and P1 at ~ 10.8%. P2, which included haplotype H1, was the most abundant and widely distributed subpopulation over time. P3 was present from 1997 to 2011. The frequency of P1 fluctuated greatly, and was the only one to undergo a drastic reduction. Its ~ 50% frequency in the 90’s fell to ~ 30% during 2003–2005 and then dropped sharply to ~ 4% in the 2006–2008 and 2009–2011 periods. In the 1990s, interestingly, P1 and P2 were the two most frequent populations (Fig. 4b). About 8% of individuals showed mixed ancestry. One mixed type consisted of P2 and P3 ancestry and was found in several, which were more frequent from 1993 to 1999 (Fig. 4c). A moderate structure was determined in Mexico compared to the global level (Additional file 5: Figure S2).

The highest *F*_ST_ values were detected between 1993–1995 and 1997–1999, and between 1997–1999 and 2000–2011 (*F*_ST_ = 0.167 and 0.153, respectively; *P* < 0.01 in both cases). The sample size was similar during these periods (Table 2).

**Table 2:** *F*_ST_ values based on *ama1*_*I-II*_ between *P. vivax* populations defined by 3-year periods in southern Mexico

Time-period:	1993–1995	1997–1999	2000–2002	2003–2005	2006–2008
1997–1999	0.1677				
2000–2002	0.0203*	0.0556**			
2003–2005	0.0580**	0.0427***	-0.0242		
2006–2008	0.0609**	0.0867***	0.0027	0.0065	
2009–2011	0.0223**	0.1530***	0.0383***	0.0439***	0.0108

*P < 0.05; **P < 0.01; ***P < 0.001

## Discussion

Due to its significant reduction in malaria cases, Mexico entered the pre-elimination phase in 2009 [50]. One of the most affected areas is Southern Mexico, a region sharing a border with Central America. The complex malaria epidemiology of this region is illustrated in the current study. For 19 years (1993–2011), the increases and decreases in *P. vivax* cases were accompanied by changes in the parameters of population genetics. The gradual reduction in *P. vivax* cases after 1999–2000 went with a reduction in the genetic diversity and the rate of mixed genotype infections, as well as an increase in linkage disequilibrium, alterations in the haplotype and subpopulation frequency, and variations in natural selection.

The 213 *P. vivax* sequences detected over 19 years represented a much higher number of *pvama1*_*I-II*_ haplotypes than that found in each of the three-year periods. Additionally, the most frequent haplotypes were present either during all or most of the study. In the majority of cases, the genetic diversity varied over time and coincided with the fluctuations in malaria cases. The greatest number of haplotypes and the highest genetic diversity (π = 0.00758), observed during 1997–1999, but the period of pre-elimination (2009–2011) witnessed the second lowest diversity (π = 0.00591) in a subgroup, and this was based on a comparable or higher number of samples than those from previous years, which indicates that this reduced value is very likely to be accurate. These results suggest that the effective population size of *P. vivax* was relatively small in southern Mexico. A previous study using microsatellites also showed that changes in the annual number of cases were accompanied by modifications in the nucleotide diversity [51].

The current study was conducted with only a few samples per year, as the focus was on the trends rather than yearly changes. The rise in malaria cases during 2006–2009 seemed not to affect the nucleotide diversity, this being similar to the previous period (2003–2005). Moreover, no genetic difference between the parasites from these two periods was suggested by the *F*_ST_ value, meaning that parasite structure was little affected.

The differentiation values can increase due to the divergence of the remaining parasites (as suggested by Volkman) [52], in regions entering the elimination phase [7], or in periods of re-emergence [13]. It was herein observed that the pre-elimination parasites were highly differentiated from parasites of uppermost transmission (1997–1999).

The detection of multiple genotype infections (MGIs) in regions with various levels of *P. vivax* transmission is common, but its rate rises in a high transmission setting [53]. In this study, there was a low-moderate proportion of MGIs and admixture (based on *pvama1*), as reported previously when using microsatellites in southern Mexico [12]. During the control phase (especially between 1993 and 1999), either MGIs or admixture rates were higher than in the elimination phase, the latter being a time when the number of MGIs and samples with admixture became negligible. This coincides with the extremely high linkage disequilibrium present in this phase.

Though the MGI rate was the greatest during the control phase, the presence of several haplotypes over time suggest that clonal transmission of *P. vivax* has been occurring in southern Mexico. The differential mosquito susceptibility to *P. vivax* strains and the geographic isolation of certain strains [12, 34] might have reduced the chances of recombination between divergent haplotypes in the region. If recombination occurred, it was between identical or very closely related genotypes, as reported by others [54]. The clonal transmission of human *Plasmodium* species occurs in both high [7, 20] and low transmission settings, the latter observed in Korea when using *pvmsp1* and *pvcsp* [55] and in Brazil with *P. falciparum* when employing *msp1* and *msp2* [56, 57].

After malaria was re-introduced in Korea, the increase in genetic diversity was the result of parasite migrations rather than the generation of new hybrid parasites [13]. Although southern Mexico shares a border with Central America, and human migration flows from South/Central to North America, the introduction of new divergent parasites was not herein demonstrated. Like Central America, the circumsporozoite vk210 and vk247 genotypes have been detected since the early 1990s in southern Mexico [58]. Consequently, the haplotype networks suggest that *P. vivax ama1*_*I-II*_ haplotypes are closely related and have recently expanded due to local adaptations [12]. This idea is supported by previous findings with *msp1* icb5-6 [35] and more recently with the genomic SNP analysis revealing that parasites from southern Mexico have recent common ancestry [59]. Such adapted, highly frequent and persistent haplotypes that are exclusive to southern Mexico might have spread as a consequence of climactic events that impeded access to communities, either by roads or rivers, thus creating impediments to the delivery of control measures.

Genes coding for diverse antigens (e.g. *pvama1*) are present in many low-frequency haplotypes that fluctuate by genetic drift, especially in populations facing a drastic demographic reduction. It cannot be ruled out that haplotypes coming from relapse cases may influence the observed changes in allele frequencies. Hypnozoite genetic reservoirs contribute to maintaining the malaria genetic pool over time [60]. Up to 1998 in southern Mexico, *P. vivax* was treated for five days with primaquine [61], which is not highly effective in preventing relapse episodes [62]. Subsequently, intermittent single doses administered to confirmed cases [60] did not avoid symptomatic and asymptomatic relapse episodes [33, 36]. In addition, gametocytes were harvested from most *P. vivax* infected samples (primary and relapse infections) [51, 63]. Another factor involved in the local development of *P. vivax* strains is the influence of climatic events, such as “El Niño” and Hurricane Stan (2005), which might have contributed to the resilience of malaria transmission despite control measures. The reports on the number of malaria cases suggest that it took about three and four years to get malaria back to a downward trend after each of these events.

Although there was a reduced haplotype pool in the pre-elimination phase, the most divergent haplotypes representing the three *P. vivax* lineages or subpopulations persisted. The yearly frequencies of *P. vivax* haplotypes underwent expansion and alternation, and the latter case was more notorious among more frequent and divergent haplotypes (e.g. H1 *vs* H8, H4 or H5). This information may be relevant for vaccine development.

There is evidence that balancing selection has maintained the polymorphism of *pvama1*_*I-II*_ [33, 36]. The haplotype alternation herein observed could have been induced by specific antibody responses [64]. It is interesting that *dN - dS* values decreased along with the decline in the number of malaria cases. Perhaps the reduction in immune individuals diminished selective pressure. Robust evidence exists that antibodies against PvAMA1 can inhibit the invasion of parasites into reticulocytes of human hosts [65, 66], and that antibody response to PvAMA1 are naturally acquired by human populations in distinct endemic regions. The influence of the antibody response has been reported in South America [67–69], the Mideast [69] and the Indian subcontinent [70, 71]. However, this does not explain the steady decrease in *dN - dS* values when malaria cases were most numerous, during 1997–1999 and 2006–2008.

## Conclusion

In summary, anti-malarial actions have rendered a gradual reduction in the number malaria cases and caused alterations in the genetic structure of the parasite in the hypoendemic region of southern Mexico, despite the fluctuations in these parameters caused by climactic events and parasite and vector characteristics. Hence, parasite elimination from the region might be possible in the near future.

## Additional files

Additional file 1: Table S1. Polymorphism and haplotypes defined for *P. vivax ama1*_*I-II*_ in southern Mexican parasites. Sixteen variable sites were detected, and 15 haplotypes (H1-H15) were resolved. (DOCX 21 kb)

Additional file 2: Figure S1. Global haplotype network of *p*v*ama1I-II*. Each circle corresponds to a haplotype (104 haplotypes) and each color represent the parasite origin, the size of the circle is proportional to the number of isolates that share the same haplotype. Single haplotypes were not considered. Solid lines connect the haplotypes, while short and perpendicular lines on the solid lines represent the number of mutational steps between two haplotypes. Small black circles indicate haplotypes not sampled or extinct. (PDF 144 kb)

Additional file 3: Table S2. Parameters of genetic diversity and recombination for *p*v*ama1*_*I-II*_ in parasite populations of different geographic origin. (DOCX 24 kb)

Additional file 4: Table S3. *F*_ST_ values between *P. vivax* populations of different geographic origin. (DOCX 23 kb)

Additional file 5: Figure S2. Population structure of *P. vivax* based on *p*v*ama1I-II*. Colors (red, blue and green) represent the resulting populations (K = 3) from Bayesian clustering analysis. A vertical bar represents each individual. (PDF 197 kb)

## Abbreviations

MGI: Mixed genotype infections
*pvama1*_*I-II*_: *P. vivax* apical membrane antigen 1 gene domains I and II
