# Supplementary material for: Temporal genetic changes in Plasmodium vivax apical membrane antigen 1 over 19 years of transmission in southern Mexico
Source: Parasit Vectors. 2017 May 2;10:217. doi: 10.1186/s13071-017-2156-y (PMC5414334; doi:10.1186/s13071-017-2156-y)
Supplement: Supplementary file 1 — Polymorphism and haplotypes defined for P. vivax ama1 I-II in southern Mexican parasites. Sixteen variable sites were detected, and 15 haplotypes (H1-H15) were resolved. (DOCX 21 kb) [file 13071_2017_2156_MOESM1_ESM.docx]

**Additional file 1**

Polymorphism and haplotypes defined for *P. vivax* *ama1_I-II_* in southern Mexican parasites. Sixteen variable sites were detected, and 15 haplotypes (H1-H15) were resolved.

|  |  | Domain I ( nt 454-747): | | | | | | | | | | Domain II (nt 748-1,155): | | | | | |
| --- | --- | --- | --- | --- | --- | --- | --- | --- | --- | --- | --- | --- | --- | --- | --- | --- | --- |
|  | n | 565 | 567 | 568 | 577 | 583 | 628 | 680 | 682 | 683 | 684 | 829 | 911 | 1056 | 1103 | 1138 | 1151 |
| Sal I |  | G | A | A | C | T | C | A | A | G | C | G | A | G | A | C | T |
| H 1  H 2  H 3  H 4  H 5  H 6  H 7  H 8  H 9  H 10  H 11  H 12  H 13  H 14  H 15 | 91  10  1  18  28  1  2  51  1  5  1  1  1  1  1 | A  **∙**  **∙**  A  A  **∙**  A  **∙**  **∙**  A  **∙**  **∙**  A  **∙**  **∙** | **T**  **∙**  **∙**  **∙**  **∙**  **∙**  **∙**  **∙**  **∙**  **∙**  **∙**  **∙**  **∙**  **∙**  **∙** | **∙**  **∙**  **∙**  C  C  **∙**  **∙**  **∙**  **∙**  C  **∙**  **∙**  C  **∙**  **∙** | **∙**  **∙**  **∙**  **∙**  **∙**  **∙**  **∙**  **∙**  **T**  **∙**  **∙**  **∙**  **∙**  **∙**  **∙** | **∙**  **∙**  **∙**  **∙**  **∙**  **∙**  **∙**  **∙**  **∙**  **∙**  **∙**  **∙**  **∙**  **∙**  **A** | **∙**  **∙**  **∙**  **∙**  **∙**  **∙**  **∙**  **∙**  **T**  **∙**  **∙**  **∙**  **∙**  **∙**  **∙** | **∙**  **∙**  **∙**  T  **∙**  **∙**  **∙**  T  **∙**  T  T  **∙**  **∙**  **∙**  T | **∙**  **∙**  **∙**  G  **∙**  **∙**  **∙**  G  **∙**  G  G  **∙**  **∙**  **∙**  G | **∙**  **∙**  **∙**  A  **∙**  **∙**  **∙**  A  **∙**  A  A  **∙**  **∙**  **∙**  A | **∙**  **∙**  **∙**  T  **∙**  **∙**  **∙**  T  **∙**  T  T  **∙**  **∙**  **∙**  T | A  A  A  A  A  A  A  A  A  A  A  **∙**  A  A  A | **∙**  **∙**  **∙**  **∙**  **∙**  **∙**  **∙**  **∙**  **∙**  **∙**  **∙**  **∙**  **∙**  **∙**  **G** | T  **∙**  **∙**  **∙**  T  **∙**  T  **∙**  **∙**  **∙**  **∙**  **∙**  **∙**  T  **∙** | **∙**  T  **∙**  **∙**  **∙**  **∙**  **∙**  T  **∙**  **∙**  **∙**  **∙**  **∙**  **∙**  T | **∙**  **∙**  **∙**  **A**  **∙**  **∙**  **∙**  **∙**  **A**  **∙**  **∙**  **∙**  **∙**  **∙**  **∙** | **∙**  C  C  C  **∙**  **∙**  **∙**  C  C  **∙**  C  **∙**  C  **∙**  C |

Sal I sequence was used as reference (XM_001615397.1); the nucleotide changes exclusive to southern Mexico are in bold; nt, nucleotides; n, number.
