# Supplementary material for: Temporal genetic changes in Plasmodium vivax apical membrane antigen 1 over 19 years of transmission in southern Mexico
Source: Parasit Vectors. 2017 May 2;10:217. doi: 10.1186/s13071-017-2156-y (PMC5414334; doi:10.1186/s13071-017-2156-y)
Supplement: Supplementary file 2 — Global haplotype network of pvama1I-II. Each circle corresponds to a haplotype (104 haplotypes) and each color represent the parasite origin, the size of the circle is proportional to the number of isolates that share the same haplotype. Single haplotypes were not considered. Solid lines connect the haplotypes, while short and perpendicular lines on the solid lines represent the number of mutational steps between two haplotypes. Small black circles indicate haplotypes not sampled or extinct. (PDF 144 kb) [file 13071_2017_2156_MOESM2_ESM.pdf]

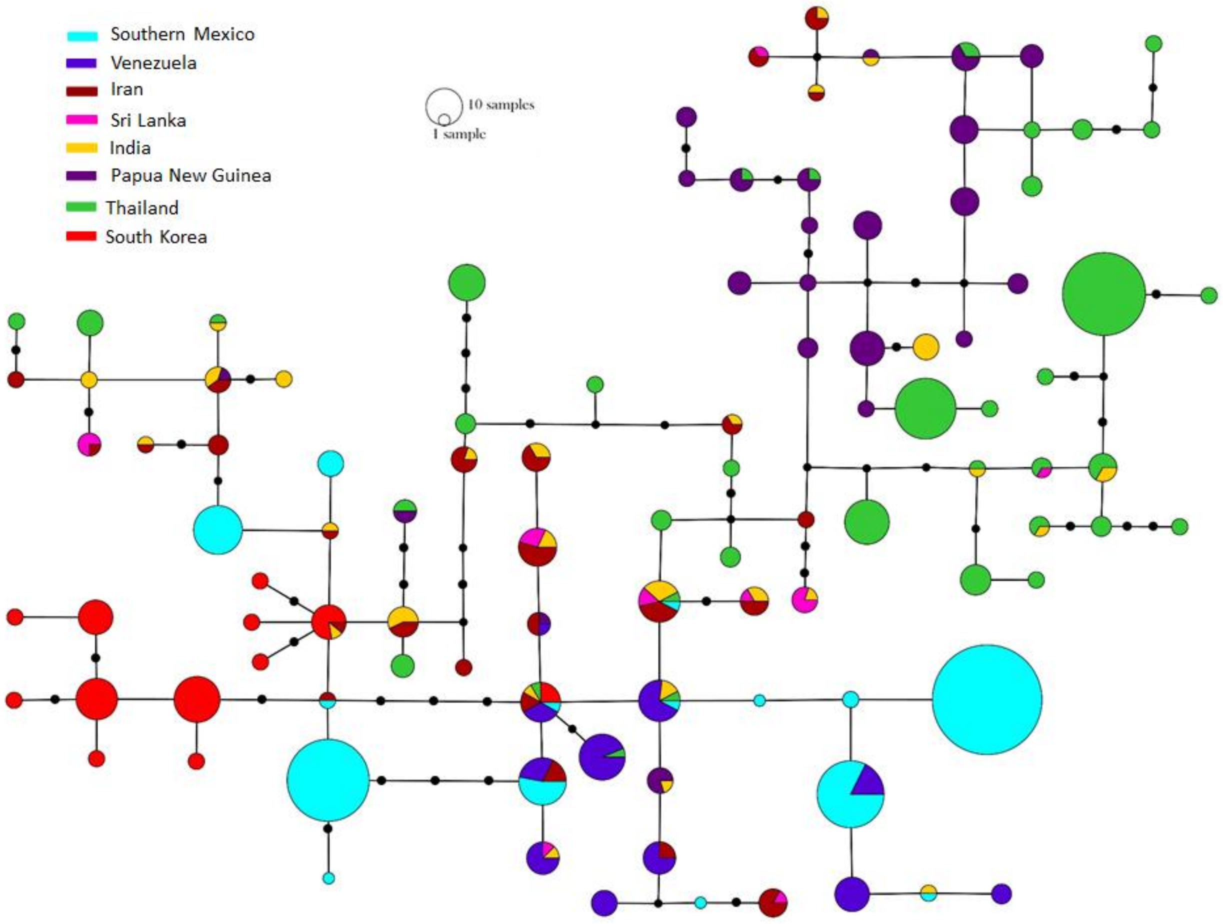

Additional file 2

Global haplotype network of *pvama1*$_{I\text{-}II}$. Each circle corresponds to a haplotype (104 haplotypes) and each color represent the parasite origin, the size of the circle is proportional to the number of isolates that share the same haplotype. Single haplotypes were not considered. Solid lines connect the haplotypes, while short and perpendicular lines on the solid lines represent the number of mutational steps between two haplotypes. Small black circles indicate haplotypes not sampled or extinct.
