# Supplementary material for: Temporal genetic changes in Plasmodium vivax apical membrane antigen 1 over 19 years of transmission in southern Mexico
Source: Parasit Vectors. 2017 May 2;10:217. doi: 10.1186/s13071-017-2156-y (PMC5414334; doi:10.1186/s13071-017-2156-y)
Supplement: Supplementary file 3 — Parameters of genetic diversity and recombination for pvama1 I-II in parasite populations of different geographic origin. (DOCX 24 kb) [file 13071_2017_2156_MOESM3_ESM.docx]

**Additional file 3**

Parameters of genetic diversity and recombination for *p*v*ama1_I-II_* in parasite populations of different geographic origin.

| **Origin** | **N** | **S** | **H** | **Hd (SD)** | **π (SD)** | **θ (SD- rl)** | **LD (R^2^)** | **Rm** |
| --- | --- | --- | --- | --- | --- | --- | --- | --- |
| **SMX** | 213 | 16 | 15 | 0.736 (0.021) | 0.0067 (0.0002) | 0.0038 (0.0002) | 0.5075 | 2 |
| **VNZ** | 73 | 11 | 15 | 0.904 (0.015) | 0.0046 (0.0002) | 0.0032 (0.0009) | 0.2077 | 3 |
| **IR** | 101 | 37 | 63 | 0.986 (0.004) | 0.0095 (0.0003) | 0.0101 (0.0016) | 0.1142 | 9 |
| **SLK** | 23 | 22 | 15 | 0.949 (0.028) | 0.0103 (0.0009) | 0.0085 (0.0018) | 0.2621 | 5 |
| **IND** | 60 | 28 | 42 | 0.983 (0.007) | 0.0092 (0.0004) | 0.0085 (0.0016) | 0.1271 | 6 |
| **PNG** | 102 | 22 | 51 | 0.976 (0.005) | 0.0074 (0.0003) | 0.0060 (0.0013) | 0.1398 | 4 |
| **THL** | 231 | 31 | 87 | 0.927 (0.012) | 0.0101 (0.0003) | 0.0073 (0.0013) | 0.1345 | 8 |
| **SK** | 66 | 26 | 16 | 0.876 (0.022) | 0.0048 (0.0004) | 0.0078 (0.0015) | 0.1521 | 1 |
| **Total** | 869 | 76 | 250 | 0.975 (0.002) | 0.0097 (0.0001) | 0.0147 (0.0017) | 0.0327 | 13 |

N; number of isolates, S; Number of polymorphic sites; H; Number of haplotypes, Hd; Haplotype diversity; π; nucleotide diversity; θ, genetic diversity; LD, linkage disequilibrium; Rm, minimal number of recombination events; SD, standard deviation. SMX, southern Mexico; VEN, Venezuela; IR, Iran; SLK, Sri Lanka; IND, India; PNG, Papua New Guinea; THL, Thailand; SK, South Korea.
