# Supplementary material for: Temporal genetic changes in Plasmodium vivax apical membrane antigen 1 over 19 years of transmission in southern Mexico
Source: Parasit Vectors. 2017 May 2;10:217. doi: 10.1186/s13071-017-2156-y (PMC5414334; doi:10.1186/s13071-017-2156-y)
Supplement: Supplementary file 4 — F ST values between P. vivax populations of different geographic origin. (DOCX 23 kb) [file 13071_2017_2156_MOESM4_ESM.docx]

**Additional file 4**

*F*_ST_ values between *P. vivax* populations of different geographic origin.

| **Country** | **SMX** | **VNZ** | **IR** | **SLK** | **IND** | **PNG** | **THL** |
| --- | --- | --- | --- | --- | --- | --- | --- |
| **SMX** |  |  |  |  |  |  |  |
| **VNZ** | 0.2234*** |  |  |  |  |  |  |
| **IR** | 0.1258*** | 0.1605*** |  |  |  |  |  |
| **SLK** | 0.1718*** | 0.1655*** | 0.0529 |  |  |  |  |
| **IND** | 0.1282*** | 0.1663*** | 0.0075 | 0.0319 |  |  |  |
| **PNG** | 0.2698*** | 0.3037*** | 0.1082*** | 0.0854*** | 0.0711*** |  |  |
| **THL** | 0.2000*** | 0.1683*** | 0.1117*** | 0.0944*** | 0.0721*** | 0.1485*** |  |
| **SK** | 0.4602*** | 0.6054*** | 0.3654*** | 0.4166*** | 0.3742*** | 0.4415*** | 0.4559*** |

* P<0.05; ** P<0.01; *** P<0.001. Based on *p*v*ama1_I-II_* SMX, southern Mexico; VEN, Venezuela; IR, Iran; SLK, Sri Lanka; IND, India; PNG, Papua New Guinea; THL, Thailand; SK, South Korea.
