# Supplementary material for: Temporal genetic changes in Plasmodium vivax apical membrane antigen 1 over 19 years of transmission in southern Mexico
Source: Parasit Vectors. 2017 May 2;10:217. doi: 10.1186/s13071-017-2156-y (PMC5414334; doi:10.1186/s13071-017-2156-y)
Supplement: Supplementary file 5 — Population structure of P. vivax based on pvama1I-II. Colors (red, blue and green) represent the resulting populations (K = 3) from Bayesian clustering analysis. A vertical bar represents each individual. (PDF 197 kb) [file 13071_2017_2156_MOESM5_ESM.pdf]

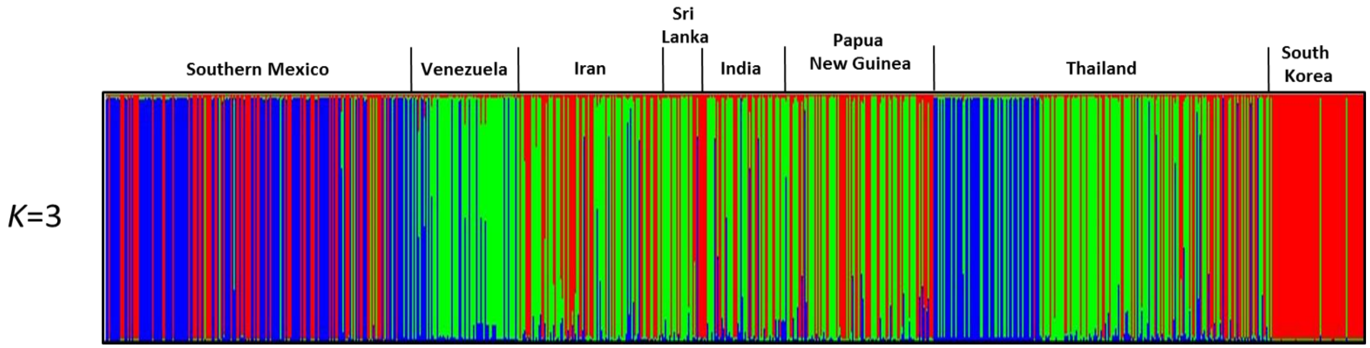

Additional file 5

Population structure of *P. vivax* based on *pvama1*$_{I\text{-}II}$. Colors (red, blue and green) represent the resulting populations (K = 3) from Bayesian clustering analysis. A vertical bar represents each individual.
